# Limitations of the L3 skeletal muscle index and the Asian Working Group for Sarcopenia 2019 consensus diagnostic criteria for sarcopenia evaluation in a case of diffuse large B‐cell lymphoma

**DOI:** 10.1002/ccr3.5949

**Published:** 2022-06-19

**Authors:** Takuya Matsunaga, Tomoko Deto, Tomoe Yamada, Takashi Yamamoto, Kimika Yamakawa, Yuta Kamiyama, Masako Morimoto, Hitoshi Seki, Fumihiko Haraguchi, Ikuko Hisatomi, Katsuya Nakanishi, Masahiro Takahashi

**Affiliations:** ^1^ Department of Medical Oncology JCHO Sapporo Hokushin Hospital Sapporo Japan; ^2^ Nutrition Management Office JCHO Sapporo Hokushin Hospital Sapporo Japan; ^3^ Rehabilitation Department JCHO Sapporo Hokushin Hospital Sapporo Japan; ^4^ Pharmaceutical Department JCHO Sapporo Hokushin Hospital Sapporo Japan; ^5^ Radiation Department JCHO Sapporo Hokushin Hospital Sapporo Japan; ^6^ Department of Anesthesiology JCHO Sapporo Hokushin Hospital Sapporo Japan; ^7^ Nursing Department JCHO Sapporo Hokushin Hospital Sapporo Japan; ^8^ Department of Pathology JCHO Sapporo Hokushin Hospital Sapporo Japan; ^9^ Department of Surgery JCHO Sapporo Hokushin Hospital Sapporo Japan

**Keywords:** AWGS 2019, DLBCL, LSMI, nutrition, rehabilitation, sarcopenia

## Abstract

Sarcopenia is an adverse prognostic factor for diffuse large B‐cell lymphoma. A case of diffuse large B‐cell lymphoma whose diagnosis, severity, and therapeutic effect of sarcopenia were difficult to determine owing to lymphoma cell infiltration into the psoas major and femoral bone marrow is reported. At presentation, the cross‐sectional area of left psoas major at L3 was enlarged owing to lymphoma cell infiltration; thus, sarcopenia evaluation was impossible by L3 skeletal muscle index. The patient was bedridden; thus, sarcopenia evaluation was impossible by the Asian Working Group for Sarcopenia 2019 consensus diagnostic criteria at presentation. At the terminal stage, she could not walk due to bilateral anterior thigh pain caused by lymphoma infiltration into femoral marrow; thus, sarcopenia evaluation was impossible by the Asian Working Group for Sarcopenia 2019 consensus diagnostic criteria. Although the L3 skeletal muscle index and the Asian Working Group for Sarcopenia 2019 consensus diagnostic criteria are representative sarcopenia evaluation systems, they cannot be used to evaluate sarcopenia in some diffuse large B‐cell lymphoma patients.

## Graphical abstract

L3 skeletal muscle index and Asian Working Group for Sarcopenia 2019 consensus diagnostic criteria are representative sarcopenia evaluation systems, and they cannot be used to evaluate sarcopenia in some patients with diffuse large B‐cell lymphoma.

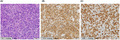

## 1 INTRODUCTION

Cancer cachexia^1^ and sarcopenia^2^ are adverse prognostic factors for diffuse large B‐cell lymphoma. The European Palliative Care Research Collaborative has proposed international definition of cancer cachexia as follows^3^: weight loss greater than 5%, or weight loss greater than 2% in individuals already showing depletion according to current body weight and height (body‐mass index <20 kg/m^2^) or skeletal muscle mass (sarcopenia). Lanic et al.^2^ demonstrated an association between quantitative skeletal muscle measurements and outcomes after R‐CHOP (rituximab, cyclophosphamide, doxorubicin, oncovin, prednisolone) treatment in patients with diffuse large B‐cell lymphoma aged over 70 years. Sarcopenia was measured by the analysis of stored computed tomography (CT) images at the third (L3) level at baseline.^2^ Skeletal muscles were assessed from a single axial slice at the L3 level because the skeletal muscles at this level are representative of the whole body.^4^ The surface of the muscular tissues was selected according to the CT Hounsfield unit. This value was normalized for stature to calculate the L3 skeletal muscle index (cm^2^/m^2^). L3 skeletal muscle index that is below 55.8 cm^2^/m^2^ for men and 38.9 cm^2^/m^2^ for women were defined as displaying sarcopenia. The Asian Working Group for Sarcopenia 2019 consensus diagnostic criteria defined sarcopenia as “age‐related loss of muscle mass, plus low muscle strength, and/or low physical performance,” without reference to comorbidity, and specified cutoffs for each diagnostic component; criteria for low appendicular skeletal muscle mass are <7.0 kg/m^2^ in men and <5.7 kg/m^2^ in women by bioelectrical impedance analysis; criteria for low muscle strength is defined as hand grip strength <28 kg for men and < 18 kg for women; criteria for low physical performance are 6‐meter walking speed <1.0 m/sec.^5^ The Asian Working Group for Sarcopenia 2019 consensus diagnostic criteria define persons with low muscle mass, low muscle strength, and low physical performance as having “severe sarcopenia.”

In this study, we report a case of diffuse large B‐cell lymphoma whose diagnosis, severity, and therapeutic effect of sarcopenia were difficult to determine owing to lymphoma cell infiltration into the psoas major and femoral bone marrow. This case report demonstrates that although L3 skeletal muscle index and the Asian Working Group for Sarcopenia 2019 consensus diagnostic criteria are representative sarcopenia evaluation systems, they cannot be used to evaluate sarcopenia in some diffuse large B‐cell lymphoma patients.

## 2 CASE DESCRIPTION

**FIGURE 1:**
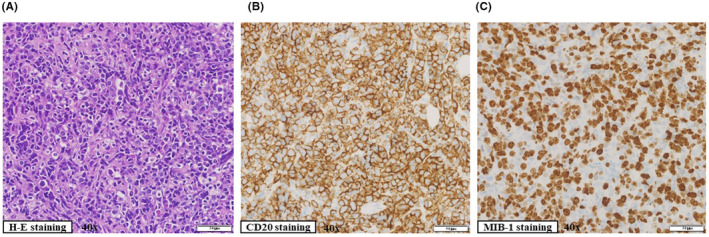
Histopathological findings of right chest wall subcutaneous mass. Immunohistochemistry was performed on paraffin‐embedded tissue. (A) Diffuse proliferation of large lymphocytes. (B) Diffuse proliferation of CD20‐positive large lymphocytes. (C) The percentage of MIB‐1‐positive cells was more than 70%

**FIGURE 2:**
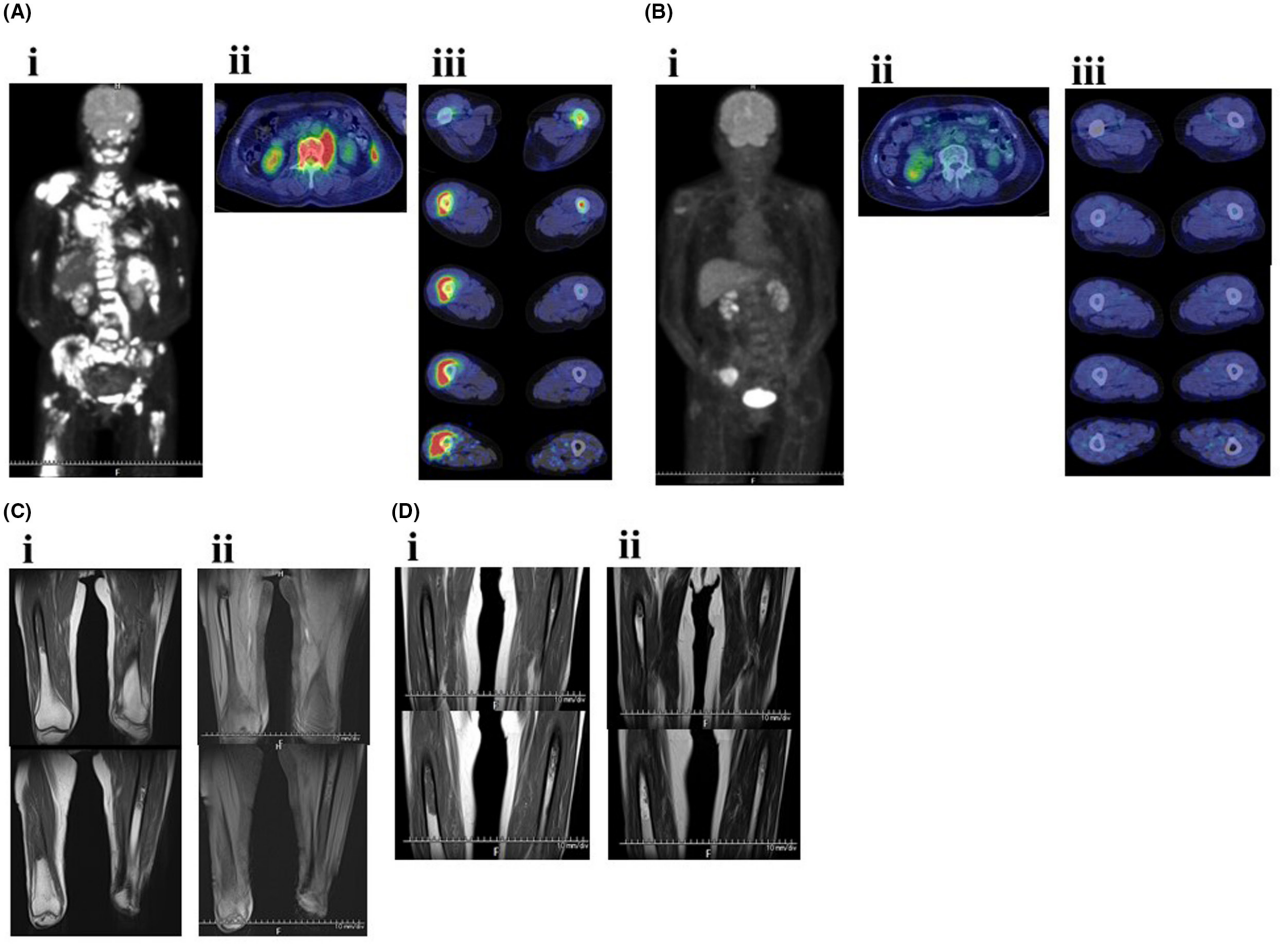
Radiological findings. (A) PET‐CT findings on June 22, 2021 ((i) systemic finding, (ii) third lumbar vertebrae finding, (iii) bilateral thighs finding). (B) PET‐CT findings on September 6, 2021 ((i) systemic finding, (ii) third lumbar vertebrae finding, (iii) bilateral thighs finding). (C) MRI finding on October 4, 2021 ((i) T1‐weighted image, (ii) T2‐weighted image). (D) MRI findings on November 4, 2021 ((i) T1‐weighted image (ii) T2‐weighted image). MRI, magnetic resonance imaging; PET‐CT, positron emission tomography‐computed tomography

An 86‐year‐old woman was referred from the Department of Oral Surgery of another hospital to our hospital in June 2021 to investigate the cause of fatigue, anorexia, body weight loss, and elevation of serum lactate dehydrogenase (LDH) and soluble interleukin‐2 receptor (sIL‐2R) levels. At presentation, she was afebrile, and physical examination revealed no skin changes, no lymphadenopathy, and no hepatosplenomegaly. A complete blood test revealed anemia (hemoglobin, 8.8 g/dl [normal range 13.6–18.3 g/dl]). An increase in serum LDH (8091 U/ml [normal range 120–245 U/ml]), sIL‐2R (1870 U/ml [normal range 122–496 U/ml]), and C‐reactive protein (CRP, 17.44 mg/dl [normal range 0.00–0.30 mg/dl]) were recognized. However, serum albumin (alb, 2.7 g/dl [normal range 3.7–5.5 g/dl]) was decreased. A needle biopsy was performed from the right chest wall subcutaneous mass and was histopathologically diagnosed as CD20‐positive diffuse large B‐cell lymphoma **(**Figure 1 **)**. Positron emission tomography‐computed tomography (PET‐CT) revealed lymphoma cell infiltration into the left psoas major and the femur **(**Figure 2A‐i,ii,iii). She was diagnosed with stage IV in the Lugano Classification. The patient's symptoms fulfilled European Palliative Care Research Collaborative international definitions of cancer cachexia^3^; her weight decreased greater than 5% within 6 months. We investigated the presence of sarcopenia. First, we used the L3 skeletal muscle index^2^ through CT imaging. However, the cross‐sectional area of the left psoas major at L3 was enlarged due to lymphoma cell infiltration; thus, sarcopenia evaluation was impossible **(**Figure 2A‐ii). Second, we used the Asian Working Group for Sarcopenia 2019 consensus diagnostic criteria.^5^ However, the patient was bedridden; thus, measurements of appendicular skeletal muscle mass by bioelectrical impedance analysis and physical performance studies (measurements of 6‐meter walking speed) were impossible.

**FIGURE 3:**
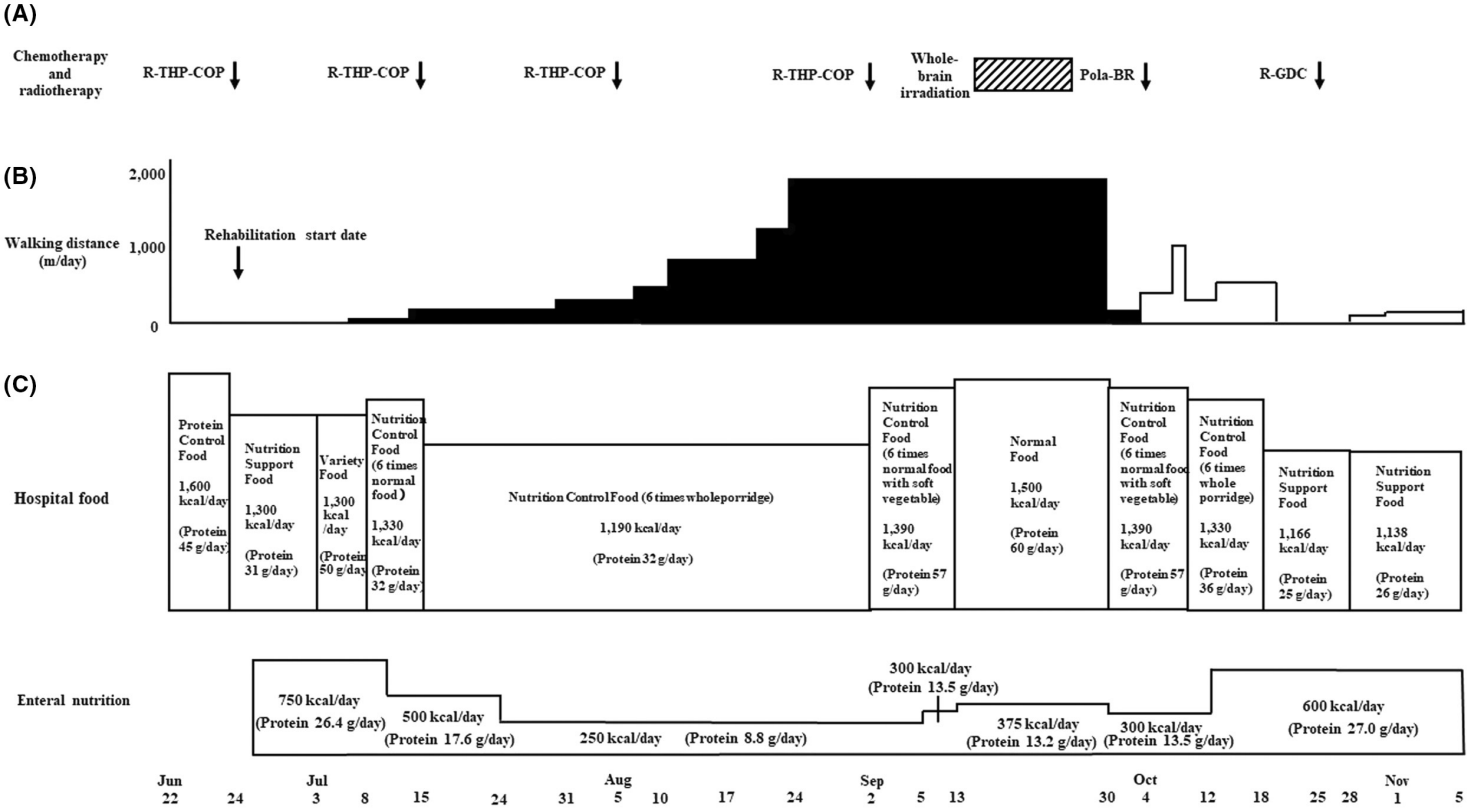
Chemotherapy, radiotherapy, walking distance, and nutritional therapy. (A) Chemotherapy and radiotherapy. 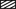, 30 Gy/ 10 fractions. (B) Walking distance. 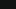, walking without walking assistance vehicle; 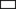, walking with walking assistance vehicle. (C) Nutrition therapy. We administered nutritional therapy using hospital food and enteral nutrition. R‐THP‐COP, rituximab, pirarubicin, cyclophosphamide, prednisolone; Pola‐BR, polatuzumab vedotin, bendamustine, rituximab; R‐GDC, rituximab, gemcitabine, dexamethasone, carboplatin

**FIGURE 4:**
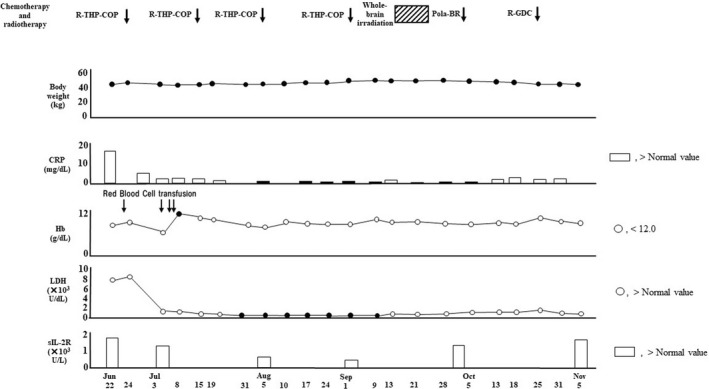
Data of body weight, CRP, Hb, LDH, and sIL‐2R. CRP, serum C‐reactive protein; Hb, blood hemoglobin; LDH, serum lactate dehydrogenase; sIL‐2R, serum soluble IL‐2 receptor. R‐THP‐COP, rituximab, pirarubicin, cyclophosphamide, prednisolone; Pola‐BR, polatuzumab vedotin, bendamustine, rituximab; R‐GDC, rituximab, gemcitabine, dexamethasone, carboplatin. 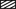, 30 Gy/10 fractions

**FIGURE 5:**
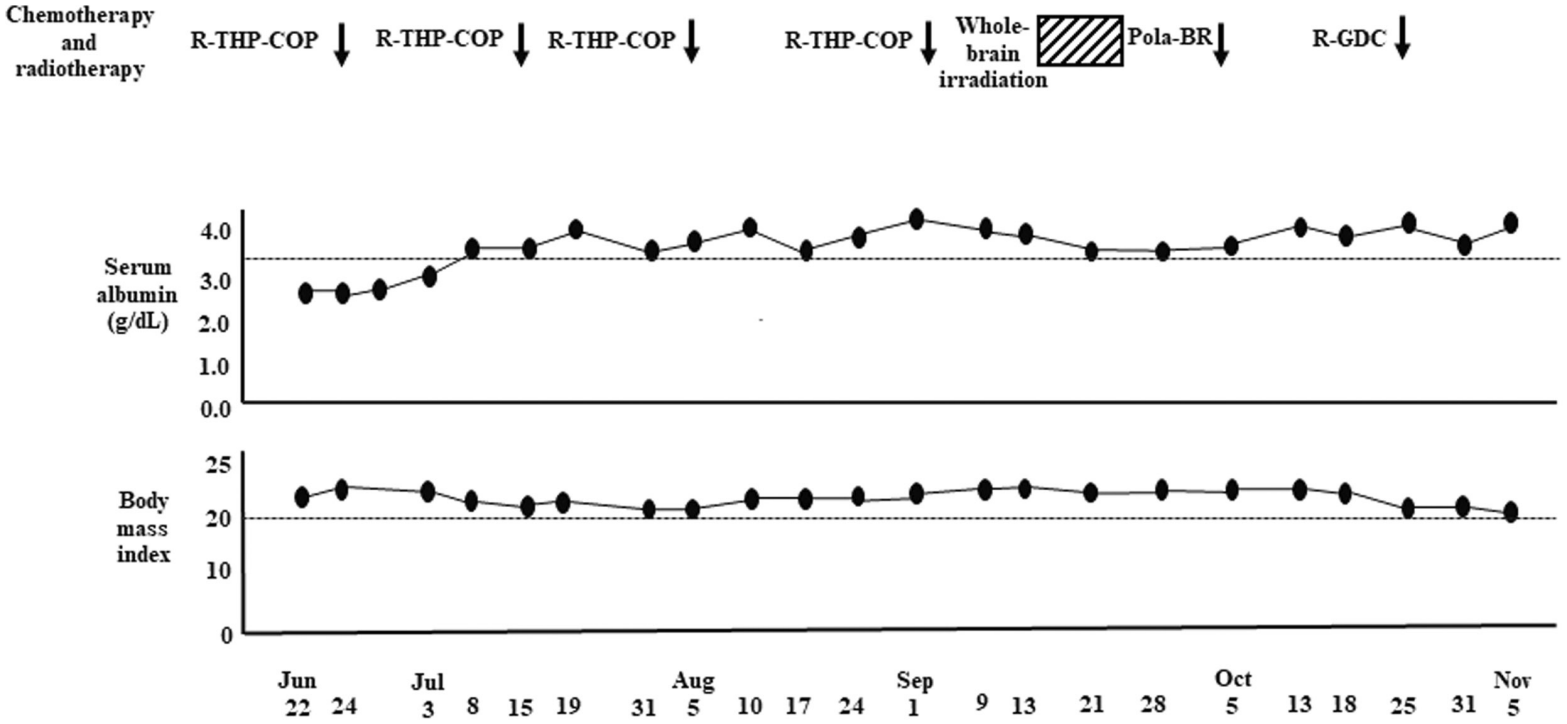
Data of nutritional status. R‐THP‐COP, rituximab, pirarubicin, cyclophosphamide, prednisolone; Pola‐BR, polatuzumab vedotin, bendamustine, rituximab; R‐GDC, rituximab, gemcitabine, dexamethasone, carboplatin. 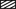, 30 Gy/10 fractions

**FIGURE 6:**
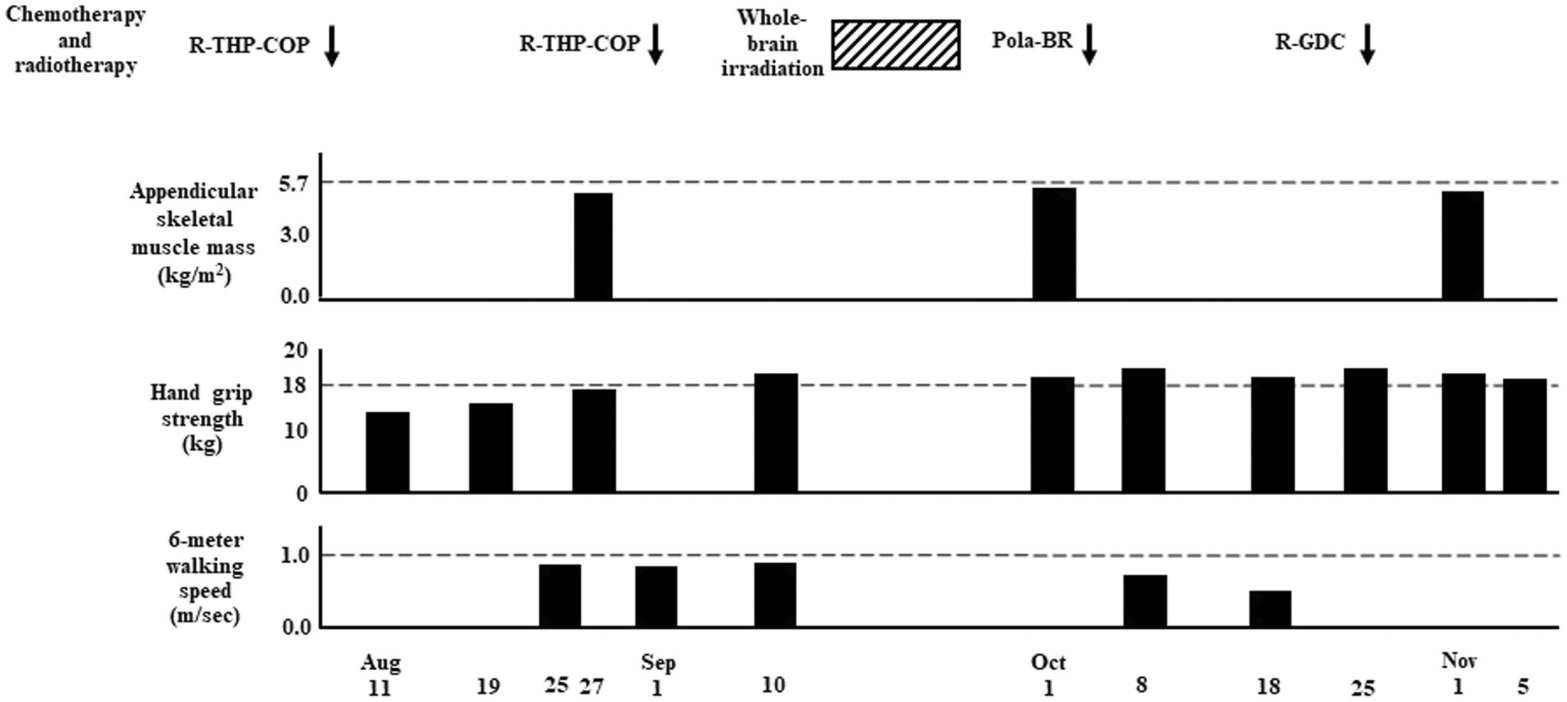
Data of Asian Working Group for Sarcopenia 2019 consensus diagnostic criteria. The criteria for low appendicular skeletal muscle mass is <5.7 kg/m^2^ in women by bioelectrical impedance analysis; criteria for low muscle strength is defined as hand grip strength <18 kg for women; criteria for low physical performance are 6‐meter walking speed <1.0 m/sec. R‐THP‐COP, rituximab, pirarubicin, cyclophosphamide, prednisolone; Pola‐BR, polatuzumab vedotin, bendamustine, rituximab; R‐GDC, rituximab, gemcitabine, dexamethasone, carboplatin. 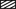, 30 Gy/10 fractions

Assuming the presence of sarcopenia, chemotherapy was initiated, and a rehabilitation program, combining exercise and nutritional intervention, was implemented **(**Figure 3 **)**. Because it was demonstrated that decreased chemotherapy dose offers a good compromise between efficacy and safety in patients with diffuse large B‐cell lymphoma aged >80 years,^6^ she was treated with attenuated chemotherapy regimens. Furthermore, she had a risk factor for febrile neutropenia at diagnosis; bone marrow involvement by lymphoma cells, and prophylactic granulocyte‐colony‐stimulating factor (G‐CSF) (150 μg/day) was administered from days 9–10 of R‐THP‐COP therapies to neutrophil recovery. After the first course of R‐THP‐COP^7^ (rituximab (375 mg/m^2^, day 1), pirarubicin (30 mg/m^2^, day 2), cyclophosphamide (500 mg/m^2^, day 2), oncovin (1.0 mg/m^2^, day 2), prednisolone (30 mg/m^2^, days 2–6)), fatigue, and serum levels of CRP, LDH, and alb were improved (Figures 4 and 5). On the contrary, body weight did not increase at all. Red blood cell transfusion was performed to address anemia during the first course of R‐THP‐COP; however, transfusion was not needed thereafter (Figure 4). We administered nutritional therapy (30–35 kcal/kg/day of energy, approximately 1 g/kg/day of protein) using hospital food and enteral nutrition. We changed the type of diet at regular intervals to suit the patient's appetite and preferences. The patient was always anorectic, and she supplemented lack of intake of hospital food with enteral nutrition. The patient trained 5 times/week with hand, lower limb, and body weight exercises (calf raises and half‐squats) as well as performed gait training. The patient could walk 1800 m/day after successful chemotherapy and nutritional rehabilitation; he was examined for sarcopenia using the Asian Working Group for Sarcopenia 2019 consensus diagnostic criteria and was found severely sarcopenic **(**Figures 3 and 6 **)**. The patient consumed nutrition control food and enteral nutrition (1440 kcal/day and 40.8 g protein/day) between July 24 and September 1, 2021. The therapeutic diet and enteral nutrition calories were increased from September 2 to overcome severe sarcopenia. PET‐CT performed on September 6, immediately after the fourth course of R‐THP‐COP, which revealed that all lesions had disappeared, except for a right iliac muscle lesion (Figures 2B‐i,ii,iii and 3). On September 10, the patient recovered her hand grip strength, but not 6‐meter walking speed (Figure 6). The patient was admitted to another hospital from September 13 to October 1 for 30 Gy/10‐fraction whole‐brain irradiation of cerebral lesions (Figure 3).

She was readmitted to our hospital on October 1 for bilateral anterior thigh pain and decreased walking distance (Figure 3). Despite analgesia, the patient's motivation decreased. Gait training was then performed using a walking assistance vehicle. After October 19, the patient's motivation further decreased; the 6‐meter walking speed could not be measured. Conversely, hand grip strength remained unchanged, and appendicular skeletal muscle mass remained low and stable (Figure 6). Magnetic resonance imaging (MRI) was performed to investigate the cause of thigh pain on October 4, 2021. The findings showed massive lymphoma cell infiltration into the bilateral femoral bone marrow (Figure 2C‐i,ii). Identical MRI findings were observed after Pola‐BR therapy^8^ (polatuzumab vedotin (1.4 mg/kg, day 2), bendamustine (68 mg/m^2^, days 2–3), rituximab (375 mg/m^2^, day 1)), and R‐GDC therapy^9^ (rituximab (375 mg/m^2^, day 8), gemcitabine (800 mg/m^2^, days 1 and 8), dexamethasone (40 mg, daily, days 1–4), carboplatin (area under the curve = 5, day 1)) on November 4, 2021 (Figures 3 and 2D‐i,ii). Since the patient had two risk factors for febrile neutropenia; prior chemotherapy and radiation therapy, the same prophylactic G‐CSF was administered. After starting chemotherapy, the serum alb level increased and remained >3.5 g/dl, and body mass index did not decrease <20 kg/m^2^ when the patient was transferred for terminal care (November 5), even when diffuse large B‐cell lymphoma worsened (Figure 5). During the hospitalization progress, body weight remained almost unchanged, serum levels of CRP, LDH, and sIL‐2R fluctuated to reflect the volume of diffuse large B‐cell lymphoma in the patient's body, and sIL‐2R levels did not decrease to normal values (Figure 4). The patient had no grade 4 neutropenia, febrile neutropenia, other grade 3–4 adverse effects during treatments. L3 skeletal muscle indexes of June 22 and September 6 were 42.5 cm^2^/m^2^ and 37.6 cm^2^/m^2^, respectively.

## 3 DISCUSSION

The patient in this case presented with body weight loss, fatigue, anemia, hypoalbuminemia, and elevated CRP levels. Therefore, the patient's symptoms and data fulfilled not only European Palliative Care Research international definition^3^ of cancer cachexia. According to Rolland et al.^10^ (i) sarcopenia is recognized as a multifactorial geriatric syndrome, (ii) cachexia is defined as a metabolic syndrome in which inflammation is the key feature, and therefore, cachexia may be an underlying condition of sarcopenia. Cancer cachexia may have also been the underlying condition of sarcopenia in our patient.

We tried nutritional rehabilitation to eliminate sarcopenia in our case. There are no established exercise and nutritional regimens for the management of sarcopenia as defined by the L3 skeletal muscle index and the Asian Working Group for Sarcopenia 2019 consensus diagnostic criteria. Furthermore, there have been no reports on how to improve sarcopenia in diffuse large B‐cell lymphoma patients. Meanwhile, nutrition therapy with exercise prescription for men with prostate cancer is thought to offer a long‐term, multi‐health benefit for managing cancer‐related fatigue.^11^ Nutritional rehabilitation might increase muscle mass and strength in older patients with sarcopenia.^12^

We administered nutrition therapy with 30–35 kcal/kg/day of energy and approximately 1 g/kg/day of protein. Energy requirements at rest are recommended as 25–30 kcal/kg/day in line with oncology‐specific European Society of Parenteral and Enteral Nutrition guidelines.^13^ To maintain and regain lean body mass and function in older people (>65 years), the PROT‐AGE study group recommends protein intake of at least 1.0–1.2 g/kg/day.^14^ Furthermore, most older adults who have acute or chronic diseases need even more dietary protein (i.e., 1.2–1.5 g/kg/day). European Society of Parenteral and Enteral Nutrition guidelines on protein intake in cancer are not directed at low muscle mass, being given as a range of 1.0–1.5 g/kg/day.^13^ On the contrary, a high‐protein diet (>1.3 g/kg/day) worsens renal function in older women with mild renal dysfunction (eGFR: 55–88 ml/min/1.73 m^2^) such as the case in our patient.^15^ We believe that body mass index and serum alb level of our patient were maintained appropriately by nutritional therapy.

Our patient trained 5 times a week with hand, lower limb, resistance training (calf raises and half‐squats), and aerobic exercises (gait training). As a result, the patient recovered handgrip strength, recovered the ability to walk a distance of 1800 m/day, and was able to maintain appendicular skeletal muscle mass.

Yoshimura et al.^16^ showed that with a program to carry out the comprehensive training, resistance exercise that is included twice a week for 3 months for 60 min might increase appendicular skeletal muscle mass and walking speed. We believe that if the patient's lymphoma cells did not infiltrate the femoral marrow, then her 6‐meter walking speed would have recovered to more than 1.0 m/sec.

Because sarcopenia is considered an adverse prognostic factor for older adult patients with diffuse large B‐cell lymphoma,^2^ hematologists should evaluate the existence and severity of sarcopenia in patients before starting therapy appropriately. Hematologists also should understand how to treat diffuse large B‐cell lymphoma patients with sarcopenia. Our chemotherapy and nutritional rehabilitation combination method might overcome sarcopenia of diffuse large B‐cell lymphoma patients.

Grade 4 neutropenia and febrile neutropenia might be evaded by attenuated chemotherapy regimens and prophylactic administration of G‐CSF. However, complete response could not be obtained using this therapeutic strategy.

Our study has limitations. We provide empirical data suggesting that a diffuse large B‐cell lymphoma patient whose diagnosis, severity, and therapeutic effect of sarcopenia were difficult to determine owing to lymphoma cell infiltration into the psoas major and femoral bone marrow. However, it is unclear what percentage of diffuse large B‐cell lymphoma patients who show the same pathology as that of our patient when using the L3 skeletal muscle index and the Asian Working Group for Sarcopenia 2019 consensus diagnostic criteria are difficult to determine sarcopenia. Furthermore, it is unknown whether sarcopenia may be difficult to determine even in diffuse large B‐cell lymphoma patients who have a different pathology from ours when using the L3 skeletal muscle index and the Asian Working Group for Sarcopenia 2019 consensus diagnostic criteria. Therefore, in the future, we would like to perform a prospective study to clarify the questions described above.

In conclusion, the L3 skeletal muscle index and the Asian Working Group for Sarcopenia 2019 consensus diagnostic criteria are representative sarcopenia evaluation systems but cannot be used to evaluate sarcopenia in some diffuse large B‐cell lymphoma patients.

In addition, these sarcopenia evaluation systems have unresolved points shown below. Although the frequency of sarcopenia in diffuse large B‐cell lymphoma is approximately 55% determined using L3 skeletal muscle index,^2^ that using the Asian Working Group for Sarcopenia 2019 consensus diagnostic criteria is not clarified. It is also unknown whether sarcopenia that meets the Asian Working Group for Sarcopenia 2019 consensus diagnostic criteria is a poor prognostic factor in diffuse large B‐cell lymphoma. The proportion of diffuse large B‐cell lymphoma patients in whom the diagnostic criteria for sarcopenia as defined by the L3 skeletal muscle index or the Asian Working Group for Sarcopenia 2019 consensus diagnostic criteria cannot be measured properly, such as in our case, has also not been clarified. We hope these unidentified points will be clarified by further clinical research.

## AUTHOR CONTRIBUTIONS

T.M. and M.T. designed this study. T.D., T.Y., T.Y., K.Y., Y.K., and I.H. contributed to gathering data. K.N. performed pathological diagnosis. T.M. wrote the main manuscript text. K.N., M.M., H.S., and F.H. analyzed data and prepared figures. All authors reviewed the manuscript.

## CONFLICT OF INTEREST

There are no conflicts of interests to report, and no funding was provided for this research.

## CONSENT

Written informed consent was obtained from the patient to publish this report in accordance with the journal's patient consent policy.

## Data Availability

All the data used in the study are available from corresponding author on reasonable request.
